# Mitomycin C-Based Hyperthermic Intraperitoneal Chemotherapy Improves Survival in Colorectal Cancer With Peritoneal Metastasis

**DOI:** 10.7759/cureus.102405

**Published:** 2026-01-27

**Authors:** Chayanit Sirisai, Hathaiwan Moungthard, Kitinut Timudom, Saipan Khunpakdee

**Affiliations:** 1 Division of Gastrointestinal and Liver Clinic, Department of Surgery, National Cancer Institute of Thailand, Bangkok, THA

**Keywords:** colorectal cancer, hyperthermic perioperative chemotherapy (hipec), mitomycin c (mmc), peritoneal metastasis, survival outcomes

## Abstract

Background: Colorectal cancer (CRC) with peritoneal metastasis is associated with poor prognosis despite advances in systemic chemotherapy and targeted therapies. Hyperthermic intraperitoneal chemotherapy (HIPEC) aims to eradicate free cancer cells within the peritoneal cavity and may improve survival outcomes. This study evaluated the efficacy and safety of mitomycin C (MMC)-based HIPEC in patients with CRC and isolated peritoneal metastasis.

Methods: Patients with CRC and peritoneal metastasis treated between November 2018 and January 2024 were retrospectively reviewed. Patients undergoing CRS followed by MMC-based HIPEC were compared with those managed without HIPEC. Clinicopathologic characteristics, perioperative outcomes, postoperative morbidity, and oncologic outcomes were analyzed.

Results: A total of 48 patients were included. The mean intraoperative Peritoneal Cancer Index was 9 (range, 2-33). Complete cytoreduction 0 was achieved in 29 patients (93.5%) who underwent CRS and HIPEC. Postoperative complications occurred in 10 patients (20.8%), with anastomotic leakage requiring reoperation in four patients (8.3%). Median overall survival was significantly longer in the HIPEC group compared with the non-HIPEC group (54.2 vs. 24.5 months, HR 0.30; 95% confidence interval [CI], 0.15-0.61; p < 0.001). The five-year overall survival rate was 47.9% in the HIPEC group vs. 4.9% in the non-HIPEC group. Median progression-free survival following HIPEC was 29.6 months (95% CI, 24.2-34.8). HIPEC was significantly associated with improved overall survival on multivariable analysis.

Conclusion: CRS combined with MMC-based HIPEC provides a significant survival benefit for selected patients with colorectal cancer and isolated peritoneal metastasis. These findings support the role of MMC-based HIPEC as an effective locoregional treatment strategy.

## Introduction

Colorectal cancer is one of the most common malignancies worldwide and represents the third most frequently diagnosed cancer in Thailand, posing a significant public health burden. Approximately 10-15% of patients with CRC present with peritoneal metastasis at initial diagnosis, a pattern of dissemination that is associated with a particularly poor prognosis [1,2]. Compared with other metastatic sites, peritoneal involvement is linked to shorter overall survival [3-5], largely due to limited drug penetration across the peritoneal-plasma barrier and reduced responsiveness to systemic chemotherapy. Despite advances in cytotoxic and targeted therapies, median survival for patients with colorectal peritoneal metastasis treated with chemotherapy alone remains unsatisfactory at 12-28 months, with a five-year survival rate less than 5% [5-7].

Over the past three decades, cytoreductive surgery (CRS) combined with hyperthermic intraperitoneal chemotherapy (HIPEC) has emerged as an aggressive locoregional treatment strategy for selected patients with peritoneal metastasis from CRC. CRS aims to achieve complete macroscopic tumor removal, while HIPEC delivers heated chemotherapeutic agents directly into the peritoneal cavity to eradicate residual microscopic disease. Multiple retrospective and prospective studies have demonstrated improved survival with CRS and HIPEC, with median overall survival extending to 40-50 months in carefully selected patients, compared with approximately 12-15 months with systemic therapy alone [8-11]. These results have led to increasing adoption of CRS and HIPEC in practice guidelines and specialized centers [12-16].

However, the role of HIPEC remains controversial. Recent randomized trials have failed to demonstrate a survival benefit with certain HIPEC regimens, particularly oxaliplatin-based protocols with short perfusion durations [11,17-19]. Moreover, CRS and HIPEC are complex procedures associated with potential morbidity, including anastomotic leakage, infectious complications, and prolonged recovery [17-19]. Consequently, appropriate patient selection, multidisciplinary evaluation, and surgical expertise are critical determinants of outcome. Prognostic factors such as the Peritoneal Cancer Index (PCI), completeness of cytoreduction (CCR), tumor biology, and patient performance status have consistently been shown to influence survival [16-22].

The present study aimed to evaluate the efficacy and safety of CRS combined with mitomycin C-based HIPEC compared with non-HIPEC treatment strategies in patients with colorectal cancer and isolated peritoneal metastasis in a real-world clinical setting. Survival outcomes, postoperative morbidity, and mortality were analyzed to further clarify the clinical value of this approach.

## Materials and methods

Patient selection 

Between November 2018 and January 2024, a total of 128 patients were evaluated at the gastrointestinal clinic. Among these, 62 patients were diagnosed with colorectal cancer with isolated peritoneal metastasis and were considered for inclusion. After applying the eligibility criteria, 48 patients were included in the final analysis. The study was approved by the institutional Ethics Committee (047_2021RC_IN730), and all data were de-identified in accordance with the Declaration of Helsinki.

Eligible patients had an Eastern Cooperative Oncology Group (ECOG) [23] performance status of 0-2, and without active significant comorbidities were eligible for inclusion. Patients with extra-abdominal metastasis, unresectable macroscopic disease, particularly the primary colon tumor, or who were unfit for major surgery were excluded. Collected variables included demographic characteristics, tumor features, perioperative treatment, histopathology, postoperative complications, length of hospital stay, recurrence, and survival outcomes.

Treatment allocation was determined through multidisciplinary team consensus, based on predefined clinical and intraoperative criteria. Patients were considered eligible for CRS with HIPEC if complete or near-complete cytoreduction (CCR-0/1) was deemed achievable, peritoneal disease burden was limited, and tumor biology and performance status were favorable. Patients who did not undergo HIPEC were managed with CRS alone or systemic therapy due to higher intraoperative PCI, inability to achieve complete cytoreduction, medical contraindications, or patient preference.

All patients underwent standardized preoperative evaluation, including laboratory testing, serum carcinoembryonic antigen (CEA) measurement, colonoscopy, and contrast-enhanced computed tomography (CT) and/or magnetic resonance imaging (MRI). Peritoneal disease burden and resectability were assessed preoperatively and confirmed intraoperatively, with PCI calculated during exploratory laparotomy. 

CRS and HIPEC treatment

An exploratory laparotomy was performed to assess disease distribution and calculate the PCI. The surgical objective was complete macroscopic tumor removal. Peritonectomy procedures were performed in regions with visible or suspected disease, and multivisceral resections were undertaken when tumor invasion was present. In female patients younger than 40 years without suspicious lesions, the uterus and ovaries were preserved. Completeness of cytoreduction was graded as CCR-0 (no residual disease), CCR-1 (residual nodules <2.5 mm), CCR-2 (2.5-25 mm), or CCR-3 (>25 mm).

Following CRS, HIPEC was administered using a hyperthermic perfusion system with semi-open, closed, or laparoscopic techniques, according to surgeon preference. Mitomycin C was administered at a dose of 35 mg/m² in normal saline over 60 minutes at a target temperature of 42-42.5°C. All intestinal anastomoses were completed prior to HIPEC. Postoperative complications within 60 days were graded according to the Clavien-Dindo classification [24]. 

Follow-up and outcome

Patients were followed every three months with clinical evaluation, laboratory tests, and tumor marker assessment. Imaging with CT or MRI was performed at six months postoperatively or after completion of chemotherapy. Overall survival was defined as the interval from diagnosis to death or last follow-up. Progression-free survival was defined as the interval from surgery to radiologic or clinical disease progression.

Statistical analysis

Patients were categorized into HIPEC and non-HIPEC groups. Descriptive statistics were used to summarize baseline characteristics. Survival outcomes were estimated using the Kaplan-Meier method and compared using log-rank testing. Univariate and multivariate analyses were performed to identify factors associated with postoperative complications and survival. A p-value < 0.05 was considered statistically significant. Statistical analyses were conducted using IBM Corp. Released 2023. IBM SPSS Statistics for Windows, Version 29. Armonk, NY: IBM Corp.

## Results

Patient characteristics

Among the 48 patients included, 31 underwent CRS and HIPEC, and 17 received non-HIPEC treatment, consisting of systemic chemotherapy with or without palliative surgery. Baseline demographic and clinicopathologic characteristics were generally balanced between groups. Detailed patient characteristics are summarized in Table 1.

**Table 1:** Demonstrates patient characteristics compared between two groups of patients CEA: carcinoembryonic antigen, CA 19-9: cancer antigen 19-9

Variable	Non HIPEC (n=17)	HIPEC (n=31)	p-value
Sex			0.763
Female	8 (47.1)	16 (51.6)	
Male	9 (52.9)	15 (48.4)	
Age (years ±SD)	53.59(±14.2)	49.5 (±12.0)	0.788
Tumor marker (g/dl^2^)			0.828
CEA	32.1 (7.3,176.7)	29.80(8.8,135.2)	
CA19-9	31.8 (8.3,181.9)	23.85 (1.9,125.8)	
Chemotherapy			
Prior chemotherapy	11 (64.7)	18 (58.1)	0.687
Perioperative chemotherapy	(0)	8 (29.0)	-
Adjuvant chemotherapy	14(82.4)	20 (64.5)	0.194
Radiotherapy			
Yes	0 (0)	3 (9.6)	-
Tumor location			
Right side	6 (35.3)	10 (32.3)	0.300
Left side	11 (64.7)	17 (54.8)	
Rectum	0 (0)	4 (12.9)	
Timing of peritoneal metastasis			0.768
Synchronous	10 (58.8)	18 (58.1)	
Metachronous	7 (41.2)	13 (41.9)	
Tumor grading			0.247
Well differentiation	0 (0)	2 (6.5)	
Moderately differentiation	13 (76.5)	17 (54.8)	
Poorly differentiation	4 (23.5)	8 (25.8)	
T stage			0.080
T2	0 (0)	1 (3.3)	
T3	3 (23.1)	17 (56.7)	
T4	10 (76.9)	12 (40.0)	
N stage			0.123
N0	4 (30.8)	4 (13.3)	
N1	2 (15.4)	8 (26.7)	
N2	7 (53.8)	9 (30.0)	
N3	0 (0)	8 (26.7)	
Follow up time (months, [range])	22.9 [3.0–110.9]	75.9 [1.2–120]	0.002

Surgical outcome

The mean intraoperative Peritoneal Cancer Index (PCI) was 9 (range, 2-33). The most common sites of peritoneal involvement were the pelvic cavity and bilateral paracolic gutters. For analytical purposes, a PCI cutoff value of 15 was applied. Patients with a PCI >15 demonstrated a higher incidence of postoperative complications; however, this association did not reach statistical significance (odds ratio [OR], 1.5; 95% confidence interval [CI], 0.21-10.79; p = 0.686).

Complete cytoreduction (CCR-0) was achieved in 29 patients (93.5%), while only one patient had a CCR score of 3. Intestinal exteriorization was required in eight patients (13.1%). Tumor characteristics of patients undergoing CRS and HIPEC are summarized in Table 2.

**Table 2:** Shows tumor characteristic of HIPEC group patients NRAS: Neuroblastoma RAS viral oncogene homolog, KRAS: Kirsten rat sarcoma viral oncogene homolog, BRCA: Breast cancer gene

Variable	HIPEC (n=31)
	N (%)
Peritoneal cancer index	
1-10	20 (64.5)
11-15	6 (19.4)
>15	5 (16.1)
Completeness of cytoreduction	
0	29 (93.5)
1	0 (0)
2	1 (3.2)
3	1 (3.2)
Genetic mutation	5 (16.1)
NRAS	1
KRAS	2
BRCA	1
Histology	
Mucinous	10 (67.7)
Signet ring cell	0 (0)
Invasion	
Lymphatic invasion	20 (87)
Perineural invasion	13 (76.5)
Visceral resection	
Liver	7 (14.6)
Spleen	12 (25.0)
Small bowel	9 (18.8)
Ostomy	8 (13.1)
Lymphatic node involvement	13 (41.9)
Ascitic cytology; Positive for malignancy	12 (38.7)
Intra-operative blood loss (ml)	600 (500,1350)
Length of hospital stay (days)	12 (9,23)
90-day mortality	0 (0)

Postoperative complications occurred in 10 patients (20.8%). Grade I complications were observed in two patients, most commonly presenting as postprandial abdominal cramping. Four patients (8.3%) developed grade II complications, including gastroparesis, all of which were successfully managed with conservative medical treatment. No hematologic or nephrologic toxicities were observed. Grade III complications occurred in four patients (8.3%), all of whom developed anastomotic leakage requiring reoperation. Notably, all patients requiring reintervention had left-sided primary tumors, and two had received prior radiotherapy. Seven patients (14.6%) had synchronous liver metastasis with a size of 1-3 cm and underwent limited liver resection. Concomitant liver resection did not increase postoperative complication rates and did not confer additional overall or progression-free survival benefit.

No chemotherapy-related toxicities, including hematologic, renal, hepatic, or cardiovascular complications, were observed. On multivariate analysis, left-sided tumor location was the only factor significantly associated with postoperative complications (hazard ratio [HR], 0.52; 95% CI, 0.35-0.79). Importantly, no postoperative mortality occurred within 90 days. Factors associated with postoperative complications are summarized in Table 3.

**Table 3:** Univariate and multivariate analyses show factors associated with complications PCI: peritoneal cancer index

Variable	Univariate analysis		Multivariate analysis	
	HR (95%CI)	p-value	HR (95%CI)	p-value
Age>60	1.37 (0.25-7.39)	0.713		
Liver resection	0.8 (0.13-5.07)	0.813		
PCI>15	1.5 (0.21-10.79)	0.686		
Left side tumor	0.52 (0.35-0.79)	0.008	0.64 (0.0-1.03)	0.025

In contrast, in the non-HIPEC group, seven patients (41.2%) were unable to complete the planned chemotherapy regimen due to poor functional status or treatment-related adverse effects, highlighting the limitations of systemic therapy alone in this patient population.

Survival outcomes and disease progression

**Figure 1:**
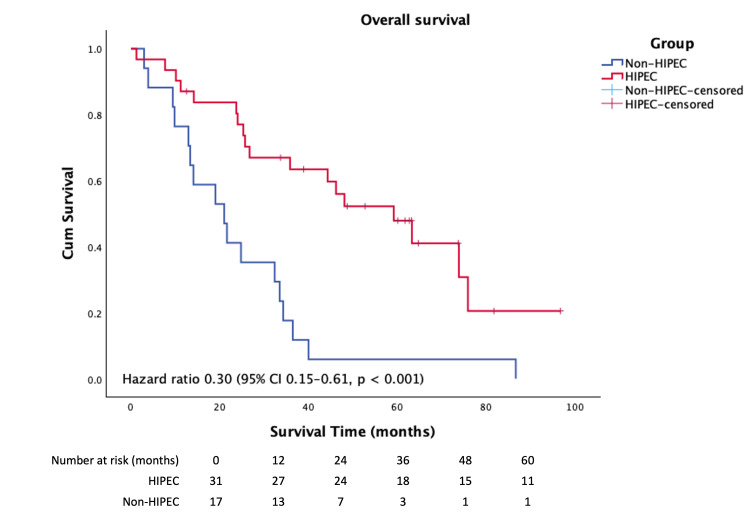
The Kaplan–Meier analysis demonstrates a significant difference in overall survival between the HIPEC and non-HIPEC groups

The mean follow-up duration was 22 months, with a significantly longer follow-up in the HIPEC group compared to the non-HIPEC group (75.9 months [range 1.2-120] vs. 22.9 months [range 3.0-110.9], p < 0.005). Median overall survival was significantly longer in the CRS and HIPEC group than in those managed without HIPEC (54.2 vs. 24.5 months; HR, 0.30; 95% CI, 0.15-0.61; p < 0.001) (Figure 1). 

The five-year survival rate was markedly higher in the HIPEC group (47.9%) compared with the non-HIPEC group (5.9%) (p < 0.001). Univariate analysis identified HIPEC, lymphovascular invasion (LVI), and perineural invasion (PNI) as factors associated with survival; however, these factors did not retain statistical significance in multivariate analysis, likely due to the limited sample size. HIPEC remained the only factor independently associated with improved survival (Table 4).

**Table 4:** Univariate and multivariate analyses show factors associated with survival CMT: chemotherapy, Rt: right, PCI: peritoneal cancer index, CCR: completeness of cytoreduction, LN: lymph node, PNI: perineural invasion, LVI: lymphatic invasion, HIPEC: hyperthermic intraperitoneal chemotherapy

Variable	Univariate analysis		Multivariate analysis	
	HR (95%CI)	p-value	HR (95%CI)	p-value
Sex	0.81 (0.226-2.9)	0.745		
Age>60	0.779 (0.192-3.160)	0.726		
Adjuvant CMT	1.806 (0.468-6.966)	0.388		
Rt side tumor	1.174 (0.299-4.615)	0.818		
PCI<15	3.429 (0.336-34.993)	0.278		
CCR 2-3	0.706 (0.040-12.433)	0.811		
LN positive	2.250 (0.504-1.053)	0.284		
PNI	10.0 (0.739-135.32)	0.057	2.23(0.72-72.86)	0.640
LVI	3.33 (1.707-6.511)	0.021	0.97 (0.09-10.59)	0.999
HIPEC	1.722 (1.277-2.323)	0.002	1.84 (0.00- 0.26)	<0.001
Liver resection	0.952 (0.174-5.228)	0.995		
Splenic resection	1.80 (0.401-8.071)	0.440		

**Figure 2:**
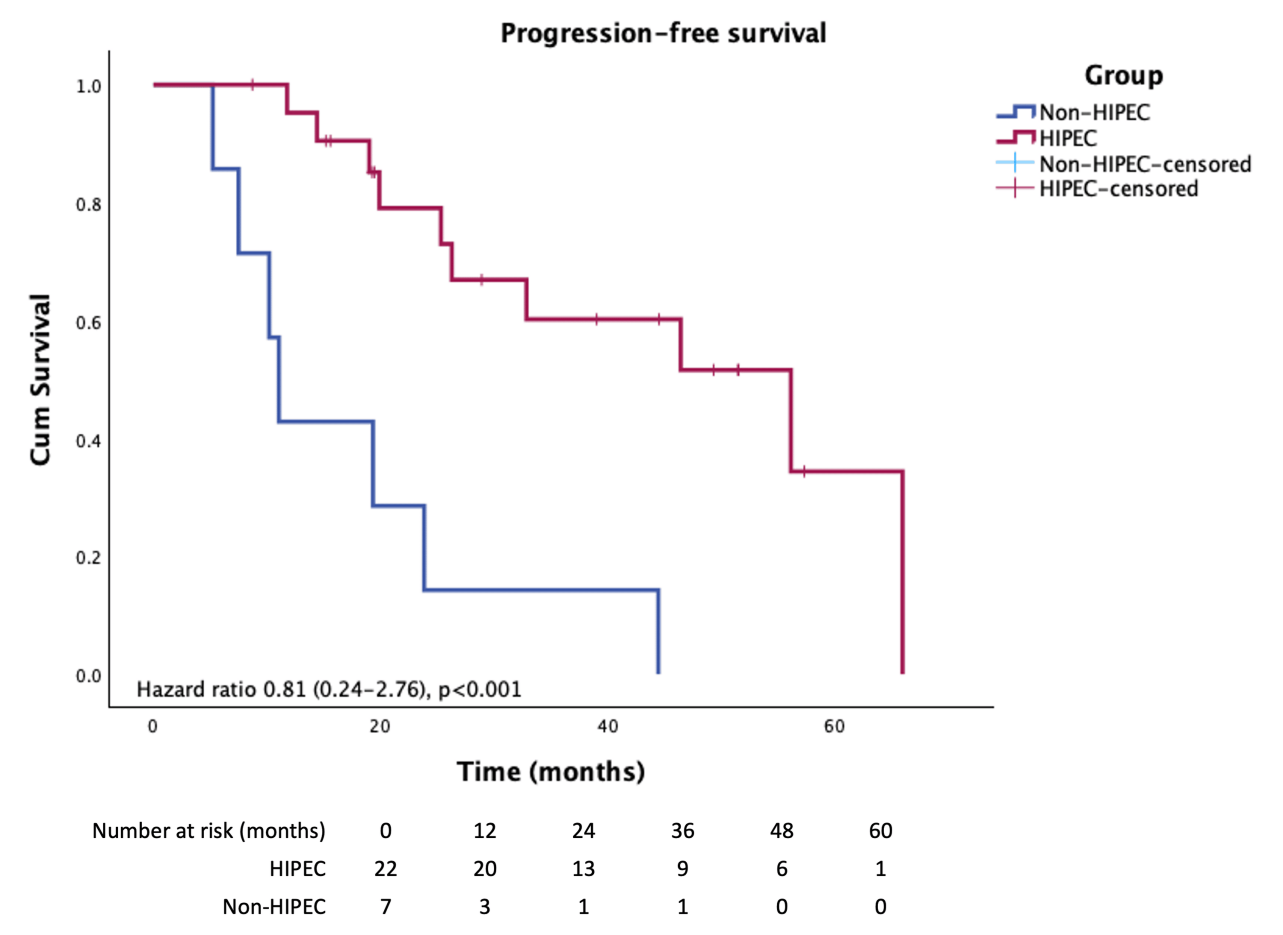
Kaplan–Meier analysis illustrating progression-free survival in patients treated with HIPEC compared with the non-HIPEC group

Mean progression-free survival (PFS) following HIPEC was 45. One month (Figure 2), whereas all patients in the non-HIPEC group experienced disease progression and death during the follow-up period, PFS was 17.4 months (HR 0.8, 95% CI 0.24-2.76). The liver was the most common site of disease recurrence after HIPEC (26.8%), followed by lung and peritoneal metastases (10.7% each). The two-year progression-free survival rate was 9.1%. The Kaplan-Meier analysis demonstrates a significant difference in overall survival between the HIPEC and non-HIPEC groups (Figure 1).

## Discussion

Colorectal cancer with peritoneal metastasis presents a challenging treatment scenario, often associated with poor outcomes. The present study demonstrates the clinical effectiveness of CRS combined with HIPEC in patients with isolated peritoneal metastasis from colorectal cancer. Patients treated with CRS and HIPEC achieved a favorable median overall survival of 54.2 months, which was substantially longer than that observed in the non-HIPEC group, where median survival was limited to 24.5 months. Although postoperative complications were observed in 20.8% of patients undergoing HIPEC, no 90-day postoperative mortality was recorded, underscoring the acceptable safety profile of this approach when performed in experienced centers. 

The results of this study are consistent with findings from the landmark randomized controlled trial conducted in the Netherlands [1,7], which reported a five-year survival rate of 45% in the HIPEC group and a median disease-specific survival of 22.2 months, compared with 12.6 months in patients receiving chemotherapy alone (p = 0.028). In addition, disease-free survival was significantly longer in the HIPEC group (12.6 months vs. 7.7 months, p = 0.020). In the current study, progression-free survival was notably prolonged, reaching 26.9 months in the HIPEC group, further supporting the oncologic benefit of this multimodal approach. In contrast, all patients in the non-HIPEC group died during the follow-up period without documented disease progression, highlighting the aggressive nature of untreated peritoneal metastasis and the limited efficacy of systemic therapy alone.

The PRODIGE 7 trial [11] failed to demonstrate a survival benefit of HIPEC in colorectal cancer with peritoneal metastasis, generating ongoing debate regarding its clinical value. A major point of controversy relates to the HIPEC protocol used in that trial, particularly the oxaliplatin-based regimen and the short duration of intraperitoneal perfusion. In the present study, MMC was administered for 60 minutes, a protocol widely adopted across multiple centers and supported by favorable oncologic outcomes [16,25-27]. This approach may partly explain the improved survival observed. Additionally, the PRODIGE 7 trial reported better survival outcomes in patients with a PCI of 11-15, which aligns with the current study’s findings that higher PCI scores were associated with increased postoperative complications, although statistical significance was not reached.

Recent literature consistently identifies PCI and CCR as critical prognostic factors influencing survival outcomes in colorectal cancer with peritoneal metastasis [16-22]. However, the present study was unable to demonstrate a statistically significant association between these variables and survival, likely due to selection bias and limited sample size. A study from Poland [21] reported that each 10-point increase in PCI was significantly associated with worse survival, reinforcing the importance of disease burden in patient selection. Notably, CCR-0 was achieved in 93.5% of patients in this study, reflecting careful multidisciplinary evaluation and substantial surgical expertise, which represent key strengths of the study.

This study also demonstrates that CRS and HIPEC can be performed with limited liver resection without increasing postoperative complications and mortality as compared to recent studies [27-29]. This highlights the feasibility of an aggressive yet carefully selected surgical approach in patients with synchronous peritoneal and limited hepatic metastases. 

This study further suggests that left-sided colon cancer is associated with an increased risk of postoperative complications, particularly anastomotic leakage. This observation differs from findings reported in a previous Korean study [30], which included a higher proportion of left-sided and rectal tumors but did not demonstrate a significant association between tumor laterality and postoperative complications. The elevated risk observed in the present cohort may be explained by anatomical and treatment-related factors, including relatively reduced vascular perfusion of the left colon and the potential detrimental effects of prior radiotherapy on tissue healing and microvascular integrity. These findings emphasize the importance of meticulous surgical technique, careful patient selection, and consideration of tumor location when planning CRS and HIPEC to minimize postoperative complications.

Nevertheless, this study was not randomized, and the decision to perform CRS and HIPEC was based on multidisciplinary assessment, likely favoring patients with more favorable prognostic characteristics, such as resectable disease and good performance status. As a result, potential selection bias cannot be excluded and may have contributed to the observed survival advantage of the CRS and HIPEC group. These findings should therefore be interpreted with caution, and prospective randomized studies are needed to better define the independent survival benefit of CRS and HIPEC.

## Conclusions

CRS combined with MMC-based HIPEC was associated with improved overall and progression-free survival in selected patients with colorectal cancer and isolated peritoneal metastasis compared with non-HIPEC management. This approach demonstrated acceptable postoperative morbidity and no 90-day mortality when performed in experienced centers. The high rate of complete cytoreduction underscores the importance of careful multidisciplinary patient selection and surgical expertise. In addition, CRS and HIPEC could be safely combined with limited liver resection, although left-sided tumors were associated with a higher risk of postoperative complications.
